# Towards Subject-Specific Strength Training Design through Predictive Use of Musculoskeletal Models

**DOI:** 10.1155/2018/9721079

**Published:** 2018-03-19

**Authors:** Michael Plüss, Florian Schellenberg, William R. Taylor, Silvio Lorenzetti

**Affiliations:** Institute for Biomechanics, ETH Zürich, Zürich, Switzerland

## Abstract

Lower extremity dysfunction is often associated with hip muscle strength deficiencies. Detailed knowledge of the muscle forces generated in the hip under specific external loading conditions enables specific structures to be trained. The aim of this study was to find the most effective movement type and loading direction to enable the training of specific parts of the hip muscles using a standing posture and a pulley system. In a novel approach to release the predictive power of musculoskeletal modelling techniques based on inverse dynamics, flexion/extension and ab-/adduction movements were virtually created. To demonstrate the effectiveness of this approach, three hip orientations and an external loading force that was systematically rotated around the body were simulated using a state-of-the art OpenSim model in order to establish ideal designs for training of the anterior and posterior parts of the *M. gluteus medius* (GM). The external force direction as well as the hip orientation greatly influenced the muscle forces in the different parts of the GM. No setting was found for simultaneous training of the anterior and posterior parts with a muscle force higher than 50% of the maximum. Importantly, this study has demonstrated the use of musculoskeletal models as an approach to predict muscle force variations for different strength and rehabilitation exercise variations.

## 1. Introduction

Detailed knowledge of the generated forces within the human musculoskeletal system provides an important step towards understanding the conditions that are required to effectively train for specific sports or undertaking targeted rehabilitation after injury or during therapy. Ideally, direct measurements of the internal loading conditions such as muscle and joint contact forces would guide such training approaches, but these are difficult to access [1, 2]. Here, while detailed datasets of kinematics and kinetics are becoming more widely available [3], such approaches are currently limited both to small populations with artificial joints as well as to only very specific sites in the human body [4–6]. As a result, musculoskeletal simulation is the primary tool for estimating internal loading conditions throughout the human body, albeit indirectly, by means of inverse dynamics and numerical optimization processes [2].

In the first steps towards understanding the interactions between kinematics and kinetics during strength training exercises [7–10], inverse dynamics approaches have been used in a subject-specific manner to enable a comparison between different exercise variations [2, 11]. In more sophisticated analyses, these approaches have been combined with muscle optimization techniques in order to compare forces in the different parts of the hamstring and quadriceps muscles between training exercises, including consideration of execution form and joint angles [10]. Performing musculoskeletal modelling requires assumptions regarding the anthropometry of the segments, shape and degree of freedom of the joints, muscular properties, and optimization criteria [12] Importantly, an in-depth understanding of the conditions under which these models are valid and able to correctly predict the internal loading conditions during squatting exercises has already been performed [13]. In an analysis using videofluoroscopy and instrumented implants, we have been able to demonstrate a flexion-dependent error in the predicted joint contact forces, but a good estimation (e.g., within 20%) over the range of 25–65° knee flexion [13]. However, despite their ability to calculate internal loading conditions throughout the musculoskeletal system, one issue that has limited the applicability of musculoskeletal modelling techniques for predicting the outcome of new exercise design is the requirement that inverse dynamics approaches are provided with known segment kinematics as a modelling input. By systematically modifying the external loading conditions, the use of these models could provide a basis for designing or improving training and rehabilitation programs for targeting specific musculoskeletal structures, thus opening a predictive capability of the approaches that has not yet been exploited.

One area that could benefit from the power of such predictive options is the focused training of hip musculature, strength deficiencies, and muscular imbalance, which, until now, has generally been investigated with respect to injury. An example of the association between adductor injury and hip strength can be seen in the frequency of adductor strains in ice hockey players, with injured players exhibiting an 18% lower hip adduction strength [14]. Importantly, the risk of adductor strain injury was shown to be almost 17 times higher in players where the adductor strength was below 80% of the abductor strength. Furthermore, recovery of the iliotibial band syndrome in long-distance runners [15] and pain [16] in subjects with retropatellar pain syndrome was improved with a gain in the strength of the hip abductor muscles. However, current strength training instructions are mostly based on the experience of the coach or physiotherapist and are rarely evidence based. This is possibly due to the complexity of the hip muscles, which include large cross-sectional areas with different parts of the same muscle active for different functional tasks, as well as different lines of action and moment arms around the joint that vary with joint angles and muscle activation. As a result, specific guidelines on how to strengthen specific parts of the hip muscles, including the direction of the external force and the joint motion, are missing in the literature. It is therefore clear that detailed knowledge of the interaction between the form of rehabilitation/strength exercise and the internal forces generated in different parts of the hip muscles could lead to an evidence-based design of training exercises for prevention and rehabilitation programs that focus on either muscular weakness or imbalance.

Compared to strength exercises for the hip muscles that include multijoint motion such as squatting, cable exercises enable an isolated movement of the hip joint as well as a specific force magnitude and direction to be applied. In addition, cable exercises enable preferential muscle force that does not affect forces and movements in other joints and is therefore a simple exercise to be simulated. Using such a pulley system, prone hip extension and straight leg raises were used in combination with musculoskeletal models to investigate the magnitude and direction of hip muscle forces [17]. Their results showed that the hip joint forces were affected by hip joint position and partially by alternations in muscle force contribution. Such studies demonstrate the importance of musculoskeletal modelling approaches to provide science-based evidence for understanding the internal muscle and force interactions towards guiding training and rehabilitation and hence positive adaptation of the tissues.

In combination with cable exercises to provide targeted force application, it therefore seems entirely plausible that musculoskeletal modelling approaches based on systematically altered kinematic and kinetic data could provide a powerful tool for designing targeted strength and rehabilitation training exercises. Therefore, the aim of this study was to evaluate the forces of hip muscles with respect to range of motion and their lengths during sagittal and frontal simulated hip strength exercises, using a musculoskeletal model driven by a systematic modification of the external force direction.

## 2. Materials and Methods

### 2.1. Description of the Exercise

**Figure 1:**
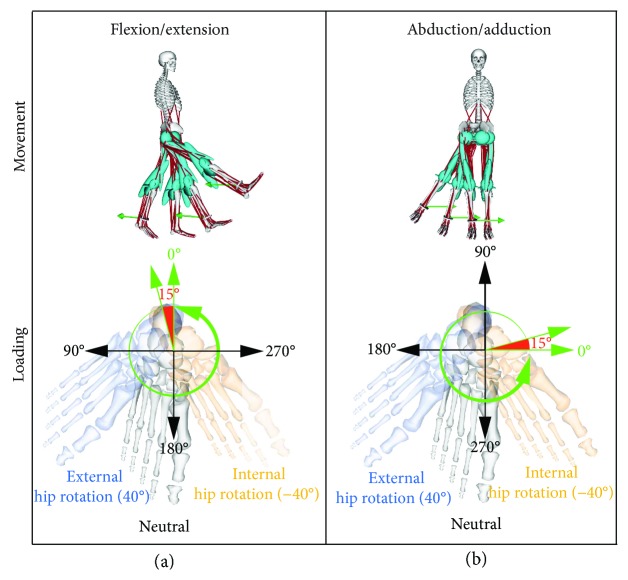
Simulation illustration (top row) of the model performing the flexion/extension (a) and the abduction/adduction (b) movements, including the 0° position of the external force (green arrow) applied to the right leg of the model. Schematic representations of the different loading conditions used in the simulation are shown in the bottom row, including rotational external force (green), which was rotated incrementally in 15° steps and three different hip rotation configurations; externally rotated (blue), neutrally rotated (grey), and internally rotated (orange).

Specific strength training exercises for the hip muscles on the cable machine were simulated (Figure 1). For these exercises, the cable is usually fixed with a strap to the shank slightly above the ankle joint and the pulley position is set as low as required in order to ensure a horizontal force vector. These exercises are single-joint and free-leg exercises. By varying the body orientation relative to the cable machine and the movement in the different anatomical planes, muscle activation changes and thus targeted muscles for strengthening can be chosen appropriately. The hip flexor and extensor muscles are then targeted by positioning the body backwards and forwards relative to the cable machine, respectively. A lateral orientation of the body and movement in the frontal plane will target the hip adductor and abductor muscles.

### 2.2. Musculoskeletal Model

The open source software OpenSim (OpenSim SimTK 3.2, Stanford, USA) was used to perform the simulation [18]. All the files required for the simulation, including motion and external force files, were created in Matlab (R2015a MathWorks, Natick, Massachusetts, USA). For the OpenSim simulation, the Arnold Lower Limb Model 2010 [19] was used. For the hip joint contact force, this model has been validated using an instrumented hip implant [20]. To apply the external loading force, a cylinder was attached rigidly to the right leg of the model to represent the ankle strap used in the strength exercises with a cable machine. The cylinder was characterized by the following dimensions: radius was set to 0.05 m, thickness 0.001 m, height 0.04 m, and mass 0.078 kg. The attachment location in the Lower Limb Model 2010 was at 0.339 m in the distal direction of the tibia coordinate system.

### 2.3. Kinematics

For kinematic inputs into the model, two different motions were created at a frequency of 110 Hz. For each, a sine-shaped movement velocity time curve was used, with a maximum movement speed of 40 degrees per second. One motion represented a hip flexion/extension (F/E) movement and was performed in the sagittal plane, while the second one characterized hip abduction and adduction (Abd/Add) and was executed in the frontal plane (Figure 1, top). The start and finish points of the F/E movement were both set at −20°-extended hip, since the Lower Limb Model 2010 was validated within this extension range only. The reversal point of the movement was set at 60° hip flexion, enabling a total range of motion (RoM) of 80°. The Abd/Add movement started with a −35° abducted hip position, where the reversal point of the movement was defined at 5° hip adduction, resulting in a 40° RoM in the frontal plane. Each limb movement was then simulated with the hip rotated at one of the three following configurations: neutral (0°), internally rotated (40°), or externally rotated (−40°) (Figure 1, bottom). In some cases, the eccentric phase and in some the concentric phase, dependent on the actual direction of the force, were at the start of the motion. The time was 6.28 s for F/E and 3.14 s for Abd/Add.

### 2.4. Kinetics

An external force with a magnitude of 100 N was applied to the centre of the attached cylinder at the shank of the model. This force represents a typical load used in a health-oriented strength training including the here-used cable exercises. In all different movements and throughout the whole cycle, the external force remained parallel to the ground. For each movement configuration, different external force directions were used to examine the influence of the position of the cable machine to the body. Starting in a dorsal direction for F/E and medially for Abd/Add simulations, the external force was then rotated incrementally by 15 degrees in a counter-clockwise direction until a complete rotation of the force was obtained, leading to 23 individual simulations (Figure 1, bottom).

### 2.5. Musculoskeletal Simulation

A quasistatic optimization was performed for all movements (F/E and Abd/Add), all hip rotations (0°, 40°, and −40°), and all kinetic parameters (force direction), to estimate the internal muscle force magnitudes, in which the sum of the squared muscle activation was minimized. This combination led to 138 individual simulations. Some simulations were run without the individual wrapping surfaces to enable successful simulation: for the F/E movement in the neutral hip position, the wrapping surface of the *M. pectineus* (PECT_at_femshaft_r) and, in the externally rotated hip position, the wrapping surface of the *M. adductor brevis* (AB_aft_femshaft_r) and the proximal part of the *M. adductor magnus* (AMprox_at_femshaft_r) were disabled due to simulation errors.

### 2.6. Evaluation of the Data

The muscle activations *A* of *M. adductor longus*, *M. adductor magnus*, *M. gluteus medius*, *M. rectus femoris*, and *M. semimembranosus* were calculated for all hip rotation configurations and external force directions as follows:

(1) $$\begin{array}{c} A=\frac{F_{act}}{F_{\max}}, \end{array}$$

where *F*_act_ is the acting muscle force and *F*_max_ is the maximal possible muscle force of the specific part of the muscle. For concentric contractions, the activation lies between 0 and 1. To properly model the anatomical characteristics, the *M. adductor magnus* and *M. gluteus medius* were included with different parts in the Lower Limb Model 2010, which were also maintained in the analysis of the parameters. 3D surface plots were then used to visualise the muscular activation, which was dependent on the joint angle as well as on the angle of the external force. Additionally, for all three hip rotation positions, the maximal activations for each external force angle were calculated and displayed in spider plots. Furthermore, the muscle lengths and the corresponding muscle activations for all three hip rotation positions were analysed at the angle of the external force where the highest activation level occurred. All data evaluation and plot generation was performed in Matlab (R2014b, MathWorks, Inc.). After initial review of the simulation data, only the muscles *M. adductor longus*, *M. rectus femoris*, *M. semimembranosus*, and *M. rectus femoris* were evaluated. Furthermore, the anterior and posterior parts of the *M. gluteus medius* (GM) were chosen for in-depth analysis, due to the fact that his muscle represents one of the major target structures of this type of cable exercises and the medial part had an activation lower than 0.5.

## 3. Results

**Figure 2:**
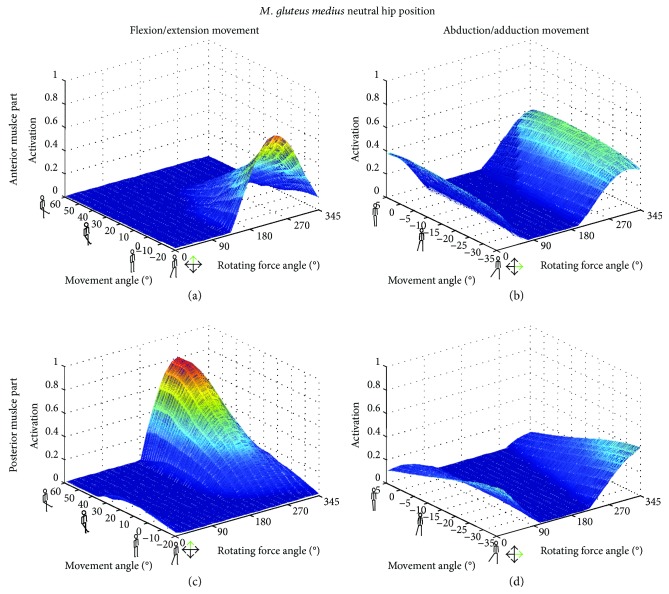
Muscle activations [0 → 1] as a function of the movement angle and all external force orientations in the neutral hip position. The anterior (a, b) and the posterior (c, d) parts of the *M. gluteus medius* (GM) are displayed for flexion/extension (a, c) and abduction/adduction (b, d) movements.

The adductor muscles' activation remained low for all loading conditions; except for *M. adductor longus*, the two rotated hip positions showed higher activities in the F/E movement than in Abd/Add. As expected, the *M. rectus femoris* exhibited a higher activation for F/E movement than for Abd/Add movement. Similar results, but in the opposite direction of the rotating external force, were observed for the *M. semimembranosus*, an antagonist of *M. rectus femoris*. Additionally, activation in the hamstrings muscles were reduced when the hip was rotated externally. An agonist/antagonist relationship was clearly visible between the anterior and posterior parts of the GM during the F/E movement with the hip in a neutral position (Figure 2). In this position, the F/E movements led to higher muscular activation compared to Abd/Add movements. The activation levels for the abductors versus the adductors remained rather equal, but this was somewhat different during F/E, where the activation seemed to increase exponentially towards a dominant maximum level. Furthermore, the activation of the anterior GM part was considerably larger within the extension range (negative angles) of the movement than within the flexion range (positive, Figure 2(a)), where the posterior part of the muscle increased in activation (Figure 2(c)).

**Figure 3 fig3:**
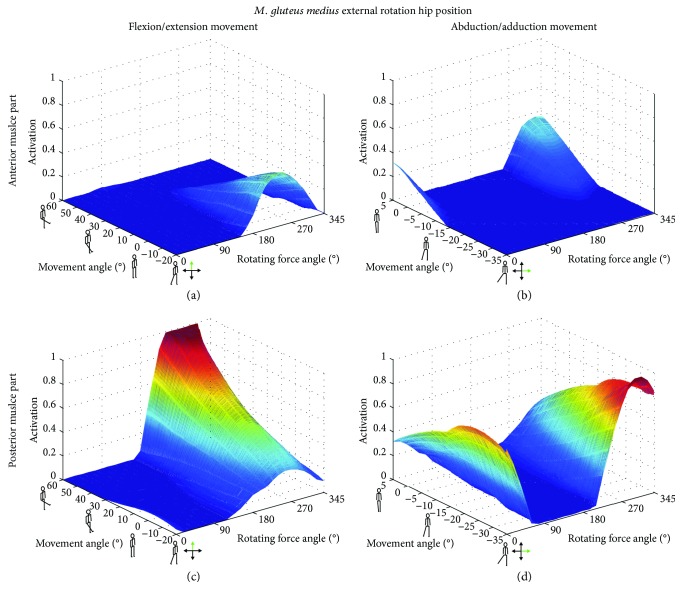
Muscle activations [0 → 1] as a function of the movement angle and external force orientations in the externally rotated hip position. The anterior (a, b) and the posterior (c, d) parts of the *M. gluteus medius* (GM) are displayed for flexion/extension (a, c) and abduction/adduction (b, d) movements.

**Figure 4:**
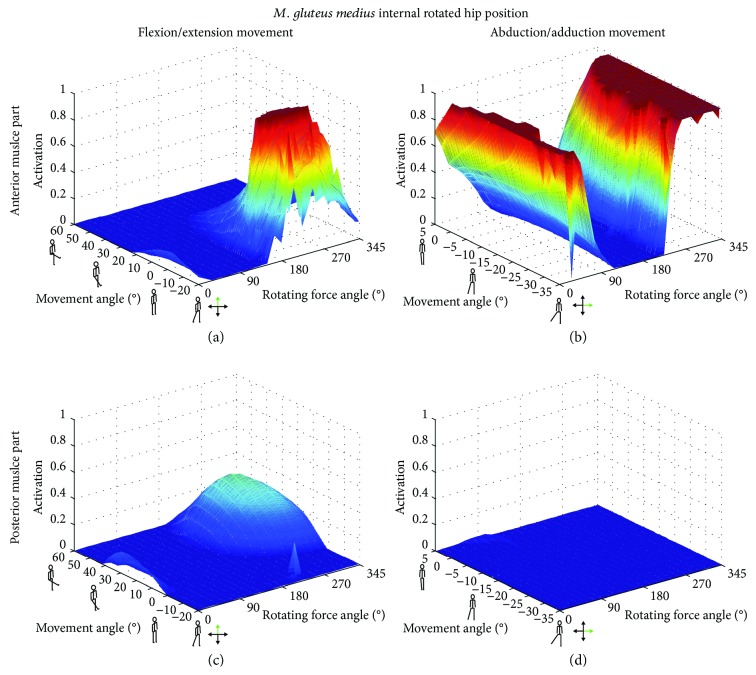
Muscle activations [0 → 1] as a function of the movement angle and external force orientations in the internally rotated hip position. The anterior (a, b) and the posterior (c, d) parts of the *M. gluteus medius* (GM) are displayed for flexion/extension (a, c) and abduction/adduction (b, d) movements.

With the hip rotated externally, the posterior part of the GM achieved a maximum activation during the flexion and abduction movements (Figures 2 [Fig fig3]–4). On the contrary, an internally rotated hip position led to maximum activation levels for the anterior part of the muscle, compared to neutral and external rotated hip positions for both movements.

**Figure 5:**
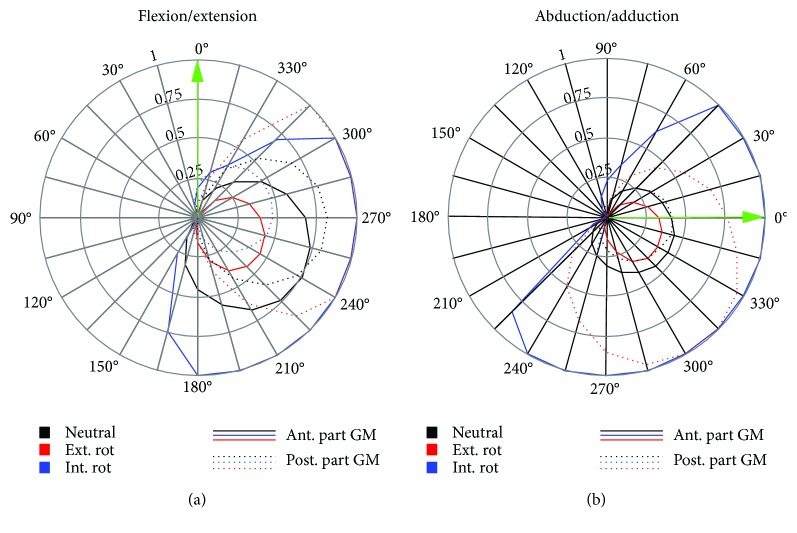
*M. gluteus medius* (GM) maximum muscle activations of the anterior (solid line) and posterior (dashed line) parts for the flexion/extension (a) and abduction/adduction (b) movements and the different hip rotation configurations: neutral (black), externally rotated (red), and internally rotated (blue). The initial external force direction (0°) of the movement is indicated by the green arrow.

**Figure 6:**
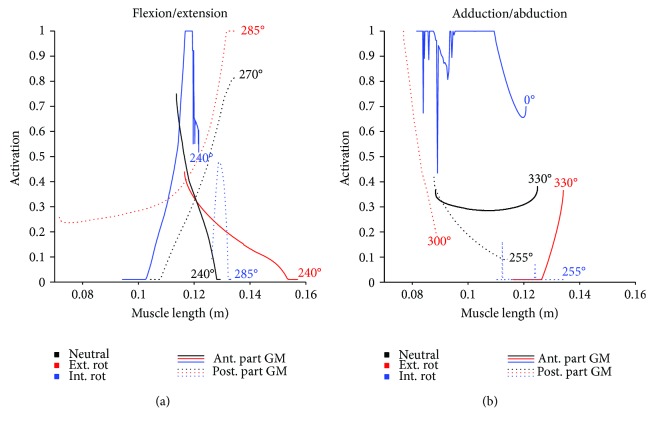
Progression of the *M. gluteus medius* (GM) muscle activations [0 → 1] with external force direction. The overall maximum activations of all force directions are shown for the anterior (solid line) and posterior (dashed line) part of the muscle as a function of the muscle length (m) for the flexion/extension (a) and abduction/adduction (b) movements, as well as for the different hip rotation configurations: neutral (black), externally rotated (red), and internally rotated (blue).

For the anterior part of the GM, maximum activation was achieved in the internally rotated hip position for external forces from 180–300° during F/E and from 0–45° as well as 240–360° for the Abd/Add movement. On the other hand, the posterior part of the GM exhibited maximum activation in the externally rotated configuration with an external force direction of 240–315° and 300–315° for the F/E and Abd/Add movements, respectively (Figure 5). While changing the rotation position of the hip had an influence on muscle length, changing the external force angles within one movement configuration had no effect on the muscle length (Figure 6). For the anterior GM, the largest muscle length changes were observed during the Abd/Add movement, while for the posterior GM, the externally rotated position caused similar activations and changes in muscle lengths in both the F/E and Abd/Add movements.

## 4. Discussion

In order to further improve rehabilitation exercises and to estimate the internal mechanical load of the specific parts of the targeted muscles, it is essential that their activation is known, with respect to the chosen movement and external loading conditions. In this study, classic hip strength and rehabilitation exercises with a F/E and an Abd/Add movement using a cable machine were simulated by means of whole-body musculoskeletal simulation with the aim to quantify muscle activation and lengths during different kinematic and kinetic configurations. To simulate the strength exercises, loading and movement patterns were generated and analysed using different directions of the cable with respect to the body, as well as using two movements with three different hip rotation positions, internally, neutrally, and externally rotated. In order to quantify the activation of the individual hip muscles and their parts, muscle activation was estimated by means of static optimization using a full body musculoskeletal model as well as targeted kinetic and kinematic conditions.

Although previous models have attempted to modify the kinematics of a joint for use in inverse dynamics modelling [21], the external forces imposed on such models are generally known (from, e.g., ground reaction force plates) and not altered. To our knowledge, this approach, where the external loading conditions were systematically varied, was used for the first time in an approach that seems to lend itself nicely towards the design of targeted training strategies through identification of the optimal movement and loading condition to specifically train a certain musculature. Here, the use of a purposefully designed hip-strengthening program can be beneficial for patients as well as athletes. Whereas it is well known that the direction of the force defines the muscle activation pattern, this work aimed also to show the importance of the hip rotation position. As an example, by including strength training exercises for abductor muscles and internal rotation in the hip, Khayambashi and coworkers [22] showed an improvement of pain and health status in women with patellofemoral pain syndrome compared to a no-exercise control group. Whole-body simulation, similar to that performed in our study, might help in the future to specifically design an efficient subject-specific workout program.

Overall, the relatively small magnitude of 100 N of the external force did not cause high activations for the *M. rectus femoris*, *M. adductor longus*, *M. semimembranosus*, and *M. adductor magnus*. Interestingly, the *M. adductor longus* showed higher activations for the F/E movement than for the Abd/Add. Here, this specific behaviour, together with increased loading, could be of interest for this muscle, since as an adductor muscle, a higher activation in the Abd/Add could be expected. Contrary to the low activation levels of the other muscles, GM showed high and alternative activations with changed kinematic and kinetic configurations. Therefore, the different parts of the GM were further analysed. Since the middle part of the GM did not achieve activations larger than 0.5, which would lead to a more efficient training stimuli, only the anterior and posterior parts were included in the in-depth analysis of the GM muscle.

In all three examined hip rotations, activation patterns of either the anterior or the posterior part was examined (Figures 2 [Fig fig3]–4). However, external or internal rotation of the hip resulted in a higher muscular activation level compared to the neutral position, which can be explained by the supportive function of these muscle parts for the hip rotation itself. Rotating the hip also influenced the length of the *M. gluteus medius* during the exercise. In order to provide the most effective training impulse to the target muscle, large muscle forces over the maximum possible change in muscle length is required [23]. For optimal training, the movement with the largest change in muscle length, together with an external force direction that causes the highest muscle force over the whole movement, should be chosen. Please note that, in this work, the muscle activation was calculated as the actual muscle force normalized by the maximum isometric muscle force. As an example, for the anterior GM part, both exercise movements with an internally rotated hip showed a high activation (Figure 4), but the Abd/Add movement also caused a greater change in muscle length (Figure 6). Therefore, based on our results, it could be recommended to train the anterior part of the GM with an internally rotated hip position using the direction of the external force in the range of the maximum activation at about 45–240°. For the posterior part, similar maximum activities and changes in muscle length were achieved with an externally rotated hip. Regarding the muscle length and the posterior part, two aspects should be considered. Firstly, the highest activation in the F/E movement occurred at large muscle length (around 0.14 m, Figure 6(a)) whereas during Abd/Add, the highest activation was observed at the shortest muscle length (around 0.08 m, Figure 6(b)). Secondly, a greater muscle length change was observed during F/E movement than during Add/Abb. Taking these two factors into account, training the posterior part should be performed in an externally rotated hip position with a F/E movement to achieve an effective training regime. In summary, our findings show the importance of properly choosing a suitable hip position, movement direction, and external force direction, in order to achieve the desired training goals. To effectively train the anterior and posterior parts of the GM, F/E movement seems to be preferable but using two different loading configurations due to the supportive functions of the two parts of the muscle in opposite hip rotation directions: for training of the anterior part, an internally rotated hip is recommended, while the highest loading for the posterior part can be achieved using an externally rotated hip.

Muscle force is known to be highly dependent on force-velocity and force-length relationships (Hill-type muscles [24, 25]). As a result, muscle activation is directly linked to the maximal isometric force capacity of the muscle, the relevant lever arm of the specific muscle, and the external loading conditions. For the sake of completeness, the force-velocity relationship will only play a minor role, since the velocity of the movement during strength training is 1.7% of the maximal shortening velocity and furthermore, the external loading changes only a few percent between the acceleration and deceleration phases [26].

Several limitations arising in this study need to be mentioned. Firstly, within the chosen model configuration and the kinematic pathways investigated in this study, only 5° of adduction in the hip could be tolerated to avoid changes in other joint angles such as the knee angle of the opposite leg. Changes in muscle activation patterns might also occur if larger adduction angles are taken into account. Secondly, the force was applied parallel to the ground and by assuming a cylinder around the ankle representing an ankle strap. This configuration would not represent the loading direction of a real cable exercise machine and could influence the results in a complex manner. Thirdly and possibly most importantly, the results of this study are based on the use of a reference and nonscaled musculoskeletal model. It is well known that the exact musculoskeletal configuration, including, for example, anatomical insertion sites [27], the definition of joint centres and axes [28–30], physiological cross-sectional areas of the muscle [31], the scaling procedure [32], and the lever arms of the muscles around the joints [33] all play critical roles on the estimation of internal loading conditions using musculoskeletal models. Particularly in the hip, relatively, little is known about the large muscles and their changing lever arms during dynamic activities. As a result, the accuracy of the muscle force and activations estimated in this study cannot be guaranteed. It is therefore imperative that further investigations using subject cohorts and, for example, electromyography (EMG) measurements, within their possibilities and limitations [34] are undertaken to test the validity of these approaches in vivo, followed by training studies to assess the relationships between these acute measures and longitudinal outcomes [35]. Further enhancement to the accuracy and reliability of musculoskeletal models would therefore only further improve the estimations of muscle forces and the specific training regime strategies. However, despite these limitations, the approaches used in this study do open perspectives for providing targeted exercise plans on a subject-specific basis or comparing muscle activations within different strength exercises by systematically varying the external force and the movement. As a result, specific exercises could be identified in order to achieve optimal loading patterns. Furthermore, by including subject-specific deficits, efficient rehabilitation regimes could be designed and even updated to follow the rehabilitation progress, if the adaptation of muscle status could be quantified.

## 5. Perspectives

Using musculoskeletal simulation and systematic variation of loading conditions, this study opens perspectives for the identification of optimal training exercises for specific muscle strengthening in rehabilitation and sports medicine. By selecting hip rotation and body positions relative to the cable exercise machine, higher muscle activation and large changes in muscle lengths can be achieved. To effectively train the anterior and posterior parts of GM muscle, two different exercises likely need to be performed. While an internally rotated hip is recommended to train the anterior part, the posterior part should be trained using an externally rotated hip. The application of these approaches in this relevant example demonstrates that precisely validated models, fed with kinematics of different exercises, could provide a powerful option to compare the effectiveness of exercises that target specific muscles. For more complex and dynamic exercises, a similar approach might be possible in the future. Here, the general procedure is similar, but the musculoskeletal model needs to enable large and extreme joint angles, individual muscle, segmental and joint properties, and subject-specific scaling.

## Supplementary Materials

Supplementary Materials The MATLAB code to generate the velocity profile as well as all the OpenSim files is available here http://www.movement.ethz.ch/data-repository.html.
